# Efficacy and safety of nab-paclitaxel plus PD-1 inhibitor in elderly patients with advanced squamous lung cancer

**DOI:** 10.1186/s12885-026-15767-z

**Published:** 2026-02-21

**Authors:** Heya Qian, Jian Jiang, Siyuan Li, Chen Ni, Yanan Chen, Yu Song, Tao Zhu, Yan Yan

**Affiliations:** 1 Department of Oncology, The Affiliated Zhangjiagang Hospital of Soochow University, Zhangjiagang, Jiangsu Province 215600 China; 2 Department of Laboratory, Centers for Disease Control and Prevention of Suzhou High-tech Zone, Suzhou, 215010 Jiangsu Province China; 3 Department of Pharmacology, The Affiliated Zhangjiagang Hospital of Soochow University, Zhangjiagang, 215600 Jiangsu Province China

**Keywords:** Advanced squamous lung cancer, Nab-paclitaxel, PD-1 inhibitor, Efficacy, Safety

## Abstract

**Background:**

This study aimed to evaluate the clinical efficacy and safety of nab-paclitaxel in combination with PD-1 inhibitors in elderly patients with advanced squamous cell lung cancer.

**Methods:**

A retrospective analysis was performed on 40 elderly patients with advanced squamous cell lung cancer treated at the affiliated Zhangjiagang Hospital of Soochow University between March 2020 and July 2024. Patients were assigned to either the chemotherapy group (*n* = 18), receiving nab-paclitaxel monotherapy, or the combination therapy group (*n* = 22), receiving nab-paclitaxel plus a PD-1 inhibitor. Treatments were administered every three weeks (per cycle). Short-term efficacy and adverse events were evaluated after every 2–3 cycles, and patients were followed up to assess the incidence of adverse reactions and clinical outcomes, including objective response rate (ORR), disease control rate (DCR), progression-free survival (PFS), and overall survival (OS).

**Results:**

Compared with the chemotherapy group, patients in the combination therapy group demonstrated improved clinical outcomes, including higher objective response rate(31.8% vs. 22.2%) and disease control rates (86.4% vs. 66.7%), as well as a longer median overall survival time (19 vs. 9 months). In addition, the combination therapy group exhibited significantly higher 6-month (100% vs. 77.8%) and 1-year (72.2% vs. 35.3%) survival rates. The most frequently observed adverse events were leukopenia, anemia, fatigue, nausea and vomiting, myalgia, cough, and interstitial pneumonia, all of which were generally manageable.

**Conclusion:**

Nab-paclitaxel combined with PD-1 inhibitors demonstrated superior short-term efficacy and an acceptable safety profile compared with chemotherapy alone in elderly patients with advanced squamous cell lung cancer.

## Background

Lung cancer remains the most frequently diagnosed cancer and the leading cause of cancer-related mortality worldwide [1]. Lung squamous cell carcinoma (LSCC) is a common subtype of lung cancer, accounting for approximately 20–30% of non-small cell lung cancer (NSCLC) cases [2]. It predominantly affects elderly male smokers and is often diagnosed at advanced stages (IIIb–IV), contributing to a high mortality rate and an estimated 450,000 deaths annually worldwide [3]. Unlike lung adenocarcinoma, which is often driven by actionable genetic mutations, LSCC is characterized by a low mutation rate and currently lacks effective molecularly targeted therapies, resulting in significantly lower survival rates [4, 5].

In clinical practice, chemotherapy remains the standard treatment for elderly patients with advanced LSCC [6]. Commonly used agents include docetaxel, paclitaxel, gemcitabine, and vinorelbine, either as monotherapies or in combination with platinum-based regimens. However, due to age-related declines in physical condition and bone marrow function, elderly patients often experience severe side effects and are unable to tolerate prolonged chemotherapy [5, 7]. The advent of immunotherapy, particularly immune checkpoint inhibitors (ICIs) targeting programmed cell death protein 1 (PD-1) and its ligand PD-L1, has revolutionized the treatment landscape for advanced LSCC. On the basis of multiple phase III clinical trials, ICIs in combination with chemotherapy have been approved as first- and second-line treatments for advanced LSCC, showing favorable outcomes regardless of PD-L1 expression [8–10]. For instance, the KEYNOTE-407 trial demonstrated that pembrolizumab combined with carboplatin and nab-paclitaxel significantly improved outcomes in treatment-naïve metastatic squamous NSCLC patients, including those aged over 75 years [11]. ICIs function by enhancing T-cell-mediated immune responses against tumor cells and are generally better tolerated than conventional chemotherapy, offering substantial clinical benefit [12, 13]. Previous clinical studies in elderly patients with non-small cell lung cancer have shown that PD-1/PD-L1 inhibitors, including pembrolizumab, nivolumab, and atezolizumab, can significantly improve overall survival (OS) whether used as first-line or later-line therapy, with an overall safety profile superior to that of conventional chemotherapy [14–17]. Trials such as KEYNOTE-189 and IMpower150 have further demonstrated that immunotherapy combined with chemotherapy remains effective in elderly patients, although the incidence of adverse events is increased, highlighting the need for careful pre-treatment assessment [18–20]. Therapeutical strategies for elderly patients with lung squamous cell carcinoma should be guided by individualized evaluation, taking into account factors such as expected survival, toxicity risk, and quality of life. Optimizing personalized immunotherapy approaches while avoiding overtreatment and improving participation of elderly patients in clinical trials is essential for effective management. Nevertheless, in older patients, immune-related adverse events such as pneumonitis, rash, and hepatic dysfunction or renal dysfunction remain challenges, making the safe and effective treatment of elderly patients with advanced LSCC an ongoing clinical concern.

Nab-paclitaxel, a novel nanoparticle albumin-bound formulation of paclitaxel, represents an important advancement in chemotherapy delivery. By binding paclitaxel to human serum albumin, nab-paclitaxel forms a unique drug delivery system that leverages albumin’s physiological roles in nutrient transport and osmotic regulation [21]. Tumor cells, with their higher metabolic demand, demonstrate increased uptake of albumin-bound substances. This allows nab-paclitaxel to more effectively target tumor tissues, resulting in enhanced drug delivery and reduced systemic toxicity [22]. Clinically, nab-paclitaxel has shown both promising antitumor efficacy and an improved safety profile [23]. Recent studies have reported that in patients with squamous cell carcinoma, the objective response rate (ORR) with nab-paclitaxel was significantly higher than that of the intention-to-treat population (41% vs. 24%, *P* < 0.001) [24]. These findings suggest that nab-paclitaxel –based regimens may offer superior therapeutic outcomes in patients with LSCC.

In 2021, China’s National Medical Products Administration approved the use of PD-1 inhibitors in combination with paclitaxel or nab-paclitaxel plus platinum as a first-line treatment for advanced LSCC lacking driver mutations [25]. However, platinum-based chemotherapy drugs have significant side effects, and elderly patients often cannot tolerate the adverse reactions of platinum drugs. Therefore, in this study, we evaluate the therapeutic efficacy and adverse reactions of Nab-paclitaxel combined with immunotherapy in elderly patients with advanced squamous cell lung cancer. Given the clinical promise of this approach, the present study aims to evaluate the short-term efficacy and safety of nab-paclitaxel in combination with PD-1 inhibitors in elderly patients with advanced LSCC. Our findings aim to provide additional evidence to support clinical decision-making and optimize treatment strategies for this vulnerable population.

## Methods

### Patient selection and retrospective grouping

This retrospective observational study was approved by the hospital’s ethics committee. All patients provided written informed consent prior to receiving chemotherapy and immunotherapy. Elderly patients with advanced pulmonary squamous cell carcinoma enrolled between March 2020 and June 2024 in the Department of Oncology at Zhangjiagang Hospital Affiliated with Soochow University. The inclusion criteria were as follows: (1) diagnosis of pulmonary squamous cell carcinoma confirmed by cytology or histopathology and immunohistochemistry; (2) clinical stage IV disease; (3) Eastern Cooperative Oncology Group (ECOG) performance status ≤ 2; (4) presence of measurable lesions based on Response Evaluation Criteria in Solid Tumors (RECIST), version 1.1 [26]; (5) no obvious contraindications to chemotherapy based on baseline hematology, cardiac, hepatic, and renal function tests; (6) age > 60 years. The exclusion criteria were as follows: (1) ECOG performance status ≥ 3; (2) severe dysfunction of vital organs such as heart, brain, liver, or kidneys; (3) allergy or intolerance to study drugs due to fever or other causes. Among the 40 patients included in this study, 33 patients received first-line treatment, while 7 patients received second-line or later (≥ 2 lines) therapy.

### Clinical retrospective study

This retrospective clinical study aimed to evaluate the efficacy and safety of nab-paclitaxel combined with PD-1 inhibitors in elderly patients (≥ 60 years) with advanced squamous cell lung cancer. The threshold of ≥ 60 years is consistent with the standard definition of older adults in China, as established by national policy and widely adopted in clinical research [27, 28]. Although higher thresholds (≥ 70/75 years) are common in international trials, our definition reflects the regional context. Furthermore, restricting enrollment to ≥ 70 years would have substantially reduced the eligible sample size, compromising the feasibility and statistical power of the analysis.

**Fig. 1:**
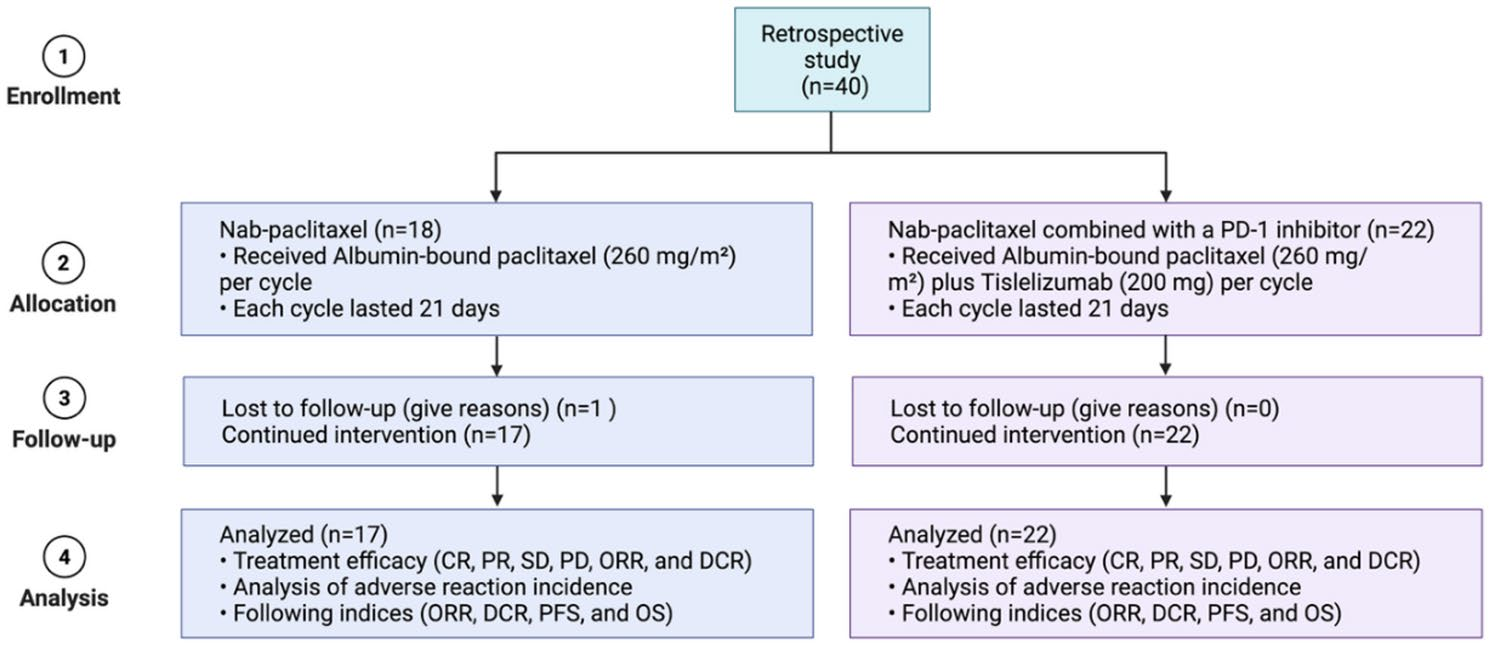
Flowchart of retrospective study participants. The number of subjects at each stage is indicated by *n*

A total of 40 elderly patients with stage IV pulmonary squamous cell carcinoma met the inclusion criteria and were enrolled, including 36 males and 4 females, with a median age of 75 years. Based on treatment regimens, patients were divided into two groups: Group 1, the chemotherapy group (nab-paclitaxel, *n* = 18), and Group 2, the combination therapy group (nab-paclitaxel combined with a PD-1 inhibitor, *n* = 22). The study flowchart is shown in Fig. 1.

In chemotherapy group: On Day 1 of each cycle, patients received Nab-paclitaxel (260 mg/m²) diluted in 100 mL of 0.9% sodium chloride via intravenous (IV) infusion. Each treatment cycle lasted 21 days, and treatment efficacy was evaluated after 2–3 cycles. Supportive care, including antiemetics, gastric protection, and anti-allergic premedication, was routinely provided.

In combination therapy group: On Day 1 of each cycle, patients received a PD-1 monoclonal antibody (Tislelizumab, 200 mg) diluted in 100 mL of 0.9% sodium chloride via IV infusion over more than 30 min. Each cycle was 21 days in length, and standard supportive care was administered as in the chemotherapy group.

If patients experienced grade III–IV chemotherapy-related adverse events, as defined by the National Cancer Institute Common Terminology Criteria for Adverse Events (NCI-CTCAE), version 5.0, the chemotherapy dose in the subsequent cycle was reduced to 75% of the previous dose.

### Short-term efficacy evaluation

Treatment efficacy was assessed according to the RECIST 1.1. Radiologic responses were categorized as follows: complete response (CR): complete disappearance of all target lesions for more than 4 weeks. partial response (PR): ≥30% reduction in the sum of diameters of target lesions. stable disease (SD): reduction < 30% or increase ≤ 20% in the sum of target lesion diameters. progressive disease (PD): >20% increase in target lesion diameters or emergence of new lesions.

In addition, the following indices were calculated: objective response rate (ORR) = (CR + PR) / total cases × 100%; disease control rate (DCR) = (CR + PR + SD) / total cases × 100%; progression-free survival (PFS), defined as the time from treatment initiation to disease progression or death from any cause; and overall survival (OS), defined as the time from treatment initiation to death from any cause.

### Adverse event monitoring

Adverse events were graded according to the NCI-CTCAE v5.0 criteria [29], which is a standardized system developed by the National Cancer Institute for classifying the severity of adverse events in clinical trials, ranging from Grade 1 (mild) to Grade 5 (death related to adverse event).

### Follow-up study

Patients were followed up via readmission, outpatient visits, or telephone. Follow-up focused on adverse events, routine blood tests, liver and kidney function, and imaging-based therapeutic evaluation. Follow-up continued until patient death or loss to follow-up. The final follow-up date was October 31, 2024.

### Statistical analysis

All statistical analyses were performed using SPSS version 21.0. Categorical data were expressed as frequency and percentage [n (%)], and comparisons between groups were analyzed using the Pearson’s Chi-squared Test or Fisher’s Exact Test. Continuous variables were presented as mean ± standard deviation and compared using the t-test. Ordinal data were analyzed using rank-sum tests. Kaplan-Meier analysis was used for survival outcomes. *P*-value < 0.05 was considered statistically significant.

## Results

### Baseline clinical characteristics

Baseline clinical characteristics of the 40 patients are summarized in Table 1. There were 18 patients in the chemotherapy group and 22 patients in the combination therapy group. No significant differences were observed between the two groups in terms of sex distribution, age, smoking history, or ECOG performance status (all *P* > 0.05), indicating comparability of baseline demographic and clinical characteristics.

**Table 1:** Comparison of baseline clinical demographics between chemotherapy and combination therapy groups

Clinical features	Chemotherapy group (*n* = 18)	Combination therapy group (*n* = 22)	Fisher’s	*P*
Sex			0.718	0.613
man	17 (94.4)	19 (86.4)		
woman	1 (5.56)	3 (13.6)		
Age (years)			0.992	0.609
80 ~ 90	3 (16.7)	3 (13.6)		
70 ~ 80	7 (38.9)	12 (54.5)		
60 ~ 70	8 (44.4)	7 (31.8)		
Smoking history			0.718	0.613
have	17 (94.4)	19 (86.4)		
not have	1 (5.56)	3 (13.6)		
ECOG grade			0.615	0.579
1	16 (88.9)	21 (95.5)		
2	2 (11.1)	1 (4.5)		

### Combination therapy shows improved disease control compared to chemotherapy

All 40 patients completed at least two cycles of antitumor therapy and were included in the short-term efficacy evaluation. No complete responses (CR) were observed in either group. In the chemotherapy group, 4 patients achieved partial response (PR), 8 patients had stable disease (SD), and 6 patients experienced disease progression (PD). In the combination therapy group, 7 patients achieved PR, 12 had SD, and 3 had PD. The objective response rate (ORR) was 31.82% (7/22) in the combination group and 22.22% (4/18) in the chemotherapy group, with no statistically significant difference (χ² = 0.41, *P* = 0.521). However, the disease control rate (DCR) was higher in the combination group at 86.4% (19/22) compared to 66.67% (12/18) in the chemotherapy group, and this difference showed statistical significance (χ² = 17.28, *P* < 0.001) (Table 2).

**Table 2:** Comparison of treatment efficacy between chemotherapy and combination therapy groups

Evaluation criteria	Chemotherapy group (*n* = 18)	Combination therapy group (*n* = 22)	Fisher’s	*P*
Complete Response (CR)	0	0		
Partial Response (PR)	4	7		
Stable Disease (SD)	8	12		
Progressive Disease (PD)	6	3		
Objective Response Rate (ORR)	22.22%	31.82%	0.41	0.521
Disease Control Rate (DCR)	66.67%	86.40%	17.28	< 0.001

### Combination therapy associated with higher 6- and 12-month survival rates in elderly patients with advanced lung squamous cell carcinoma

All 40 patients were successfully followed up. As of the final follow-up date (October 31, 2024), the follow-up duration ranged from 3 to 43 months. Among the 18 elderly patients in the chemotherapy group, one patient was lost to follow-up after receiving treatment for an abdominal aortic dissection at an external hospital. At the end of follow-up, 10 of the 22 elderly patients in the combination therapy group were alive. Among them, 4 patients had overall survival and progression-free survival exceeding 12 months, while the remaining 6 had both OS and PFS exceeding 6 months.

The median OS was 9 months (95% CI: 0.219–1.024) in the chemotherapy group and 19 months (95% CI: 0.9764–4.564) in the combination therapy group. Median OS was significantly longer in the combination therapy than in the chemotherapy alone (19.0 months vs. 9.0 months). The Gehan–Breslow–Wilcoxon test, which gives greater weight to early events, revealed a statistically significant difference between the two groups (χ² = 5.581, *P* = 0.018). In contrast, the log-rank test, which assumes proportional hazards and is more sensitive to late differences, did not demonstrate a statistically significance difference (χ² = 0.929, *P* = 0.335). Collectively, these data indicate that combination therapy confers a clinically meaningful early survival benefit.

**Fig. 2:**
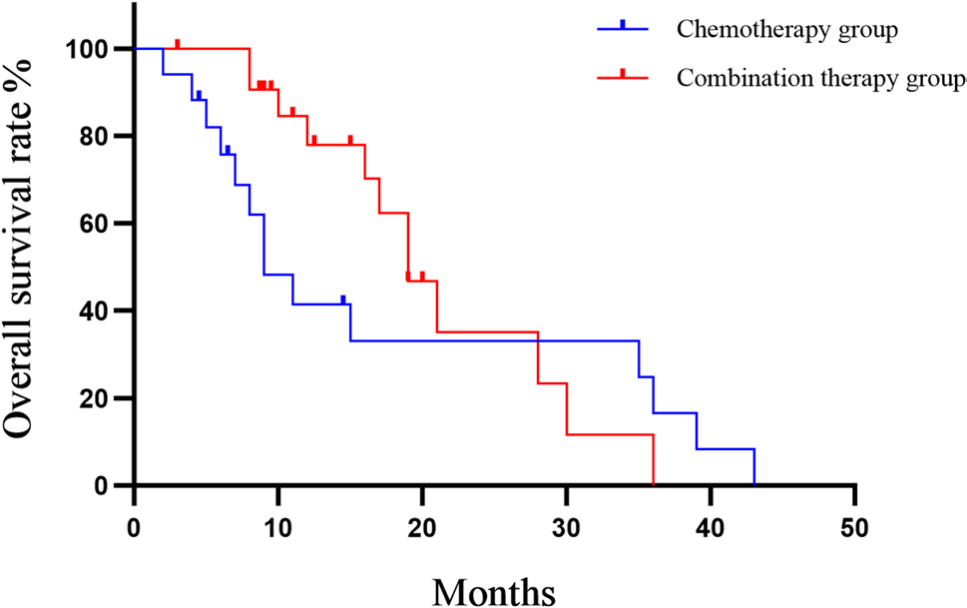
Kaplan–Meier survival curves of OS comparing patients receiving chemotherapy (*n* = 18) versus those receiving combined therapy (*n* = 22)

We further analyzed the short-term survival rate. The 6-month survival rate was significantly higher in the combination group at 100% (22/22) compared with 77.8% (14/18) in the chemotherapy group (χ² = 5.296, *P* = 0.021). Similarly, the 12-month survival rate was also significantly higher in the combination group (72.2%, 13/18) compared to the chemotherapy group (35.3%, 6/17) (χ² = 4.667, *P* = 0.031). Although the 18-month survival rate was higher in the combination group (50%, 8/16) than in the chemotherapy group (23.5%, 4/17), the difference was not statistically significant (χ² =2.420, *P* = 0.120). Interestingly, the 2-year survival rate was lower in the combination group (21.4%, 3/14) than in the chemotherapy group (23.5%, 4/17), though this difference was also not statistically significant (χ² = 0.019, *P* = 0.891) (Fig. 2).

**Fig. 3:**
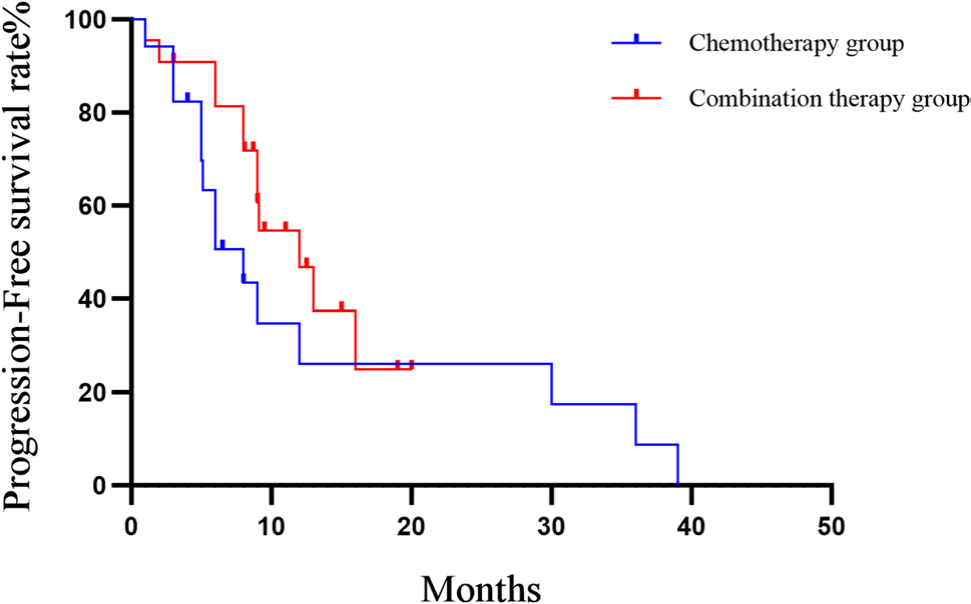
Kaplan–Meier survival curves of PFS comparing patients receiving chemotherapy (*n* = 18) versus those receiving combined therapy (*n* = 22)

The median PFS was 12.0 months (95% CI: 0.683–3.243) in the combination therapy group versus 8.0 months in the chemotherapy group (95% CI: 0.308–1.441). However, neither the log-rank test (χ² = 1.432, *P* = 0.231) nor the Gehan-Breslow-Wilcoxon test (χ² = 2.895, *P* = 0.089) showed a statistically significant difference between the two groups (Fig. 3). Thus, the observed numerical prolongation of PFS with combination therapy did not reach statistical significance.

Our findings suggest that elderly patients with advanced lung squamous cell carcinoma receiving combination therapy experienced significantly higher 6-month and 12-month survival rates compared with those receiving chemotherapy. 

### Adverse events were generally mild and manageable, with no significant differences between treatment groups

Adverse events were assessed in all 40 elderly patients with advanced lung squamous cell carcinoma. Hematologic toxicities mainly included leukopenia and anemia, with most events graded as mild to moderate (grade Ⅰ–Ⅱ). In the chemotherapy group, three patients experienced grade Ⅲ leukopenia, while only one patient in the combination therapy group experienced grade III leukopenia. No grade Ⅳ hematologic toxicities were observed in either group.

Non-hematologic adverse events included fatigue, nausea, vomiting, myalgia, hepatic dysfunction or renal dysfunction, cough, and interstitial pneumonia, the majority of these events were grade I–II. Most adverse events were manageable, and no statistically significant differences in the overall incidence of adverse events were observed between the two groups (Table 3).

**Table 3:** Comparative analysis of adverse events incidence between chemotherapy and combination therapy groups

Adverse events	Chemotherapy (n= 18)			Combined treatment (*n* = 22)			Z	*P*
	Level 1-2	Level 3-4	total	Level 1-2	Level 3-4	total		
Leukocytes/granulocytes decreased	7	3	10	12	1	13	0.222	0.824
Hemoglobin reduction	2	0	2	0	0	0	1.584	0.113
Hepatic insufficiency	0	0	0	1	0	1	0.905	0.366
Renal inadequacy	1	0	1	0	0	0	1.106	0.269
Cough	1	0	1	2	0	2	0.417	0.677
Muscular soreness	6	0	6	7	1	8	0.197	0.844
Nausea and vomiting	4	0	4	5	0	5	0.286	0.775
Chronic fibrous pneumonia	0	0	0	2	0	2	1.296	0.195

Interstitial pneumonia was observed only in the combination therapy group (2 cases, grade Ⅰ–Ⅱ), with an incidence of 9.1%. In addition, one patient in the combination group developed grade III fatigue and myalgia. Further evaluation revealed a markedly decreased adrenocorticotropic hormone (ACTH) level (3.14 pg/mL), suggesting PD-1 inhibitor–associated cortisol suppression. The patient was treated with oral prednisone (5 mg once daily) for one month, which led to symptomatic improvement. Anti-tumor treatment was temporarily discontinued during this period.

Overall, adverse events in both treatment groups were generally manageable, and no unexpected safety signals were observed.

## Discussion

Elderly patients with lung squamous cell carcinoma often present with multiple comorbidities, compromised pulmonary function, reduced chemotherapy tolerance, and limited physical reserves. Moreover, the lack of targetable driver mutations in LSCC limits the benefit of targeted therapies in this population, making chemotherapy and immunotherapy the mainstays of treatment [30]. Consequently, optimizing therapeutic strategies for elderly patients with advanced LSCC remains an important clinical challenge.

The Elderly Lung Cancer Vinorelbine Italian Study phase III trial demonstrated that chemotherapy provided a survival advantage over best supportive care in elderly patients with advanced non-small cell lung cancer [31, 32]. However, the optimal choice between single-agent and combination chemotherapy has yet to be clearly established. In a large phase III study by Gridelli et al. [33], 707 elderly patients were randomized to receive single-agent gemcitabine, vinorelbine, or combination therapy. The study showed no significant differences in response rate, progression-free survival, or overall survival among the groups, although combination therapy was associated with a higher incidence of adverse events. On this basis, current ASCO guidelines recommend single-agent chemotherapy as the preferred approach in elderly patients with advanced NSCLC.

Our study demonstrated that the ORR in the combination therapy group was higher than that in the chemotherapy group, although the difference was not statistically significant. The DCR in the combination therapy group was significantly higher than that in the chemotherapy group, with a statistically significant difference. The 6-month and 12-month survival rates were also higher in the combination therapy group. Therefore, for elderly patients with advanced squamous cell lung cancer, treatment with nab-paclitaxel combined with a PD-1 inhibitor results in a higher disease control rate and more pronounced benefits within the first year, leading to longer survival time.

Nab-paclitaxel is a guideline-recommended first-line agent for squamous cell carcinoma and offers several advantages over traditional paclitaxel formulations. These include improved tumor drug delivery via albumin-mediated transport, higher intratumoral drug concentrations, better tolerability, and ease of administration without the need for solvent-based premedication [34].

In our study, combination therapy with nab-paclitaxel and a PD-1 inhibitor demonstrated numerically higher disease control rate and objective response rate compared with chemotherapy, although these differences were not statistically significant. Importantly, the 6-month and 1-year survival rates were significantly higher in the combination group, suggesting that the addition of immunotherapy may confer a survival benefit within the first year of treatment in elderly patients with advanced LSCC.

Adverse events were generally comparable between the two groups and predominantly mild to moderate (grade Ⅰ–Ⅱ). Grade Ⅲ leukopenia occurred in three patients in the chemotherapy group and only one patient in the combination group, supporting the favorable hematologic safety profile of nab-paclitaxel. A small number of immune-related adverse events (irAEs) were observed in the combination group, including one case of grade Ⅲ fatigue and myalgia associated with decreased ACTH levels, which improved with oral prednisone and temporary interruption of anticancer therapy. In addition, two cases of interstitial pneumonia were also observed exclusively in the combination group, potentially related to immune checkpoint inhibition. Given the smoking history and baseline pulmonary impairment in these patients, the contribution of pre-existing lung conditions cannot be excluded. However, clinicians should remain alert to immune-related toxicities, particularly in patients receiving PD-1 inhibitor–based combination therapy. Combination therapy may be considered for patients with good performance status and no significant pulmonary comorbidities, whereas nab-paclitaxel monotherapy may be a safer option for those with poorer functional status or pre-existing lung disease.

For elderly patients with poor pulmonary function, chronic bronchitis, emphysema, or a history of chronic cough, immunotherapy combined with chemotherapy should be used with caution because of the increased risk of immune-related adverse events. Furthermore, most patients in this retrospective cohort did not undergo PD-L1 testing due to financial limitations, which is a limitation of our study. Nevertheless, current evidence supports PD-L1 testing in elderly patients with driver mutation–negative, advanced, and unresectable LSCC, as those with PD-L1 expression ≥ 50% are more likely to benefit from immune checkpoint inhibitors. Tumor mutational burden (TMB) may also serve as an additional predictive biomarker.

Interestingly, four female patients in this cohort were never-smokers. According to current clinical guidelines, molecular testing for EGFR, ALK, ROS1, BRAF V600E, and MET exon 14 skipping alterations is recommended in never-smokers, patients with limited biopsy specimens, or those with mixed histologic features, in order to inform subsequent treatment decisions.

In summary, our findings suggest that nab-paclitaxel combined with a PD-1 inhibitor may improved short-term survival compared with nab-paclitaxel monotherapy in elderly patients with advanced LSCC, with manageable safety profiles in both groups. For patients with an ECOG performance status of 0–1 and no significant pulmonary comorbidities, combination therapy may be a reasonable therapeutic option. Conversely, in patients with an ECOG performance status of 2 or underlying pulmonary disease, nab-paclitaxel monotherapy may be considered. Given the distinct physiological characteristics of elderly patients, including increased susceptibility to myelosuppression, fatigue, and organ dysfunction, careful monitoring for treatment-related adverse events is essential throughout the course of therapy. This study has several limitations. First, the relatively small sample size may have limited the statistical power to detect differences between treatment groups and may affect the generalizability of our findings. Second, given the retrospective study design, treatment allocation was not randomized, and potential confounding factors could not be fully controlled. In addition, selection bias cannot be entirely excluded. Accordingly, the findings should be interpreted with caution and require confirmation in larger, prospective, multicenter studies.

## Conclusions

In conclusion, this retrospective analysis indicates that nab-paclitaxel combined with a PD-1 inhibitor may offer improved short-term efficacy compared with nab-paclitaxel monotherapy in elderly patients with advanced LSCC, with an acceptable safety profile. These findings support further prospective investigation to better define the role of combination therapy in this patient population. 

## Data Availability

All data generated in this analysis are available from the first author.

## Abbreviations

LSCC: Lung squamous cell carcinoma
NSCLC: Non-small cell lung cancer
PD-1: Programmed cell death protein 1
PD-L1: Programmed ell death ligand 1
ORR: Objective response rate
ECOG: Eastern Cooperative Oncology Group
RECIST: Response Evaluation Criteria in Solid Tumors
NCI-CTCAE: National Cancer Institute Common Terminology Criteria for Adverse Events
CR: Complete response
PR: Partial response
SD: Stable disease
PD: Progressive disease
ORR: Objective response rate
DCR: Disease control rate
PFS: Progression-free survival
OS: Overall survival
ACTH: Adrenocorticotropic hormone
IrAEs: Immune-related adverse events
TMB: Tumor mutational burden
